# Time Estimation of Polymer Translocation through Nano-Membrane

**DOI:** 10.3390/polym14102090

**Published:** 2022-05-20

**Authors:** Maria-Alexandra Paun, Vladimir-Alexandru Paun, Viorel-Puiu Paun

**Affiliations:** 1 School of Engineering, Swiss Federal Institute of Technology (EPFL), 1015 Lausanne, Switzerland; maria_paun2003@yahoo.com; 2 Division Radio Monitoring and Equipment, Section Market Access and Conformity, Federal Office of Communications OFCOM, 2501 Bienne, Switzerland; 3 Five Rescue Research Laboratory, 75004 Paris, France; vladimir.alexandru.paun@ieee.org; 4 Department of Physics, Faculty of Applied Sciences, University Politehnica of Bucharest, 060042 Bucharest, Romania; 5 Academy of Romanian Scientists, 050094 Bucharest, Romania

**Keywords:** polymer, nanometric pores, nucleic acids, free energy, translocation time

## Abstract

In this paper, the charged polymer escapement phenomenon, via a little hole of nano-metric dimensions arranged in a constitutive biological membrane, is studied. We will present the case of the transport process of an ideal polymer in a 3-dimensional extended region separated by a fine boundary named membrane in a free energy barrier attendance. Additionally, the general translocation time formula, respectively, the transition time from the cis area to the trans area, is presented. The model for estimation of the likelihood, designated by *P*(*x*, *t*), as a macromolecular chain of lengthiness equal to *x*, to be able to pass by the nanopore in escape period *t*, was optimized. The longest-lasting likely escape time found with this model is indicated to be *t_p_* = 330 μs. Thus, the results obtained with the described formula are in good agreement with those announced in the specialized literature.

## 1. Introduction

The dynamics of polymer translocation through nanopores has been one of the most active research areas during the past few decades.

**Figure 1:**
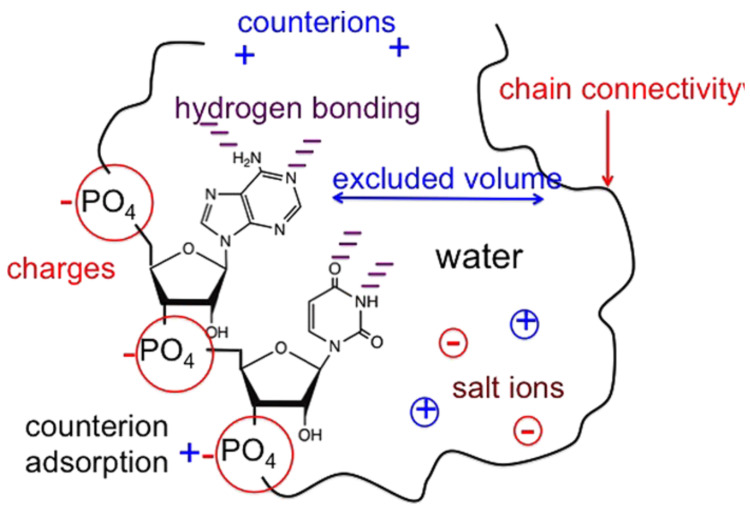
Charged polymer and chain connectivity schema.

The charges and polymer chain connectivity, counter ions, ions of salt, excluded volume repercussion, primordial hydrogen bond, and water organized themselves and contributed to the arrangement and functions of these polyelectrolytes, Figure 1.

About what a natural polyelectrolyte is, we can say the following: polyelectrolytes can be either synthetic or natural. Nucleic acids, proteins, teichoic acids, some polypeptides, and some polysaccharides are examples of natural polyelectrolytes.

The major advantages of polymers in polymeric nanoparticles are manifold. Of these, we can quote the most important advantages offered by the polymeric nanoparticles, which include the following: (1) provide controlled release to the desired site, (2) provide stability to labile molecules (e.g., proteins), and (3) provide the ability to modify surfaces with ligands for stealth and targeted drug delivery purposes [1,2].

We will refer here only to the disadvantages of polymer membranes and disadvantage of polymers as membrane materials, respectively. In nanofiltration (NF), for example, we are dealing with NF membranes whose pores vary from 0.01 μm to 0.001 μm.

Against such solutions, many polymer-based membranes (which comprise the majority of membrane materials used today) can dissolve, swell, or weaken to the extent that their lifetimes become unacceptably short or their selectivities become unacceptably low. What the disadvantages of the membrane structure are, is the second problem.

In Figure 1, a general scheme of a charged polymer and a polyelectrolyte is presented.

This perspective on the proposed subject approached here is a notional breviary of various heuristic progress related to polymer dimension and electrical charge, as well as the personal way of approaching and treating the problem in question.

Let us now present the latest techniques for obtaining nano-membranes and nanopores that are dimensionally compatible with them.

Transport properties of membranes are closely related to morphological properties like surface porosity and variation of their inner pore structure. Scanning electron microscopy (SEM), as well as transmission electron microscopy (TEM), are powerful tools to characterize the microscopical pore structure of membranes in a qualitative manner. In order to provide more quantitative data on surface and cross-sectional pores, computer image analysis can be used.

More precisely, the porosity profiles describe the local pore variation within the membrane quantitatively, generating additional information about the functionality of the filtration process [2].

The reactive ion etching technology or the electron beam from a transmission electron microscope (TEM) can be used to construct nanopores on silicon nitride (SiN_x_) and silicon oxide (SiO_2_) membranes. These two approaches demonstrate great repeatability and have been widely used in fabricating nanopores, but the method of choice is to drill them using an electron beam in a TEM. Scanning electron microscopy (SEM) is another technique where only milligram quantities of material may be used to determine particle size, shape, and texture [3]. In SEM, a fine beam of electrons scans across the prepared sample in a series of parallel tracks. The electrons interact with the sample and produce several different signals, which can be detected and displayed on the screen of a cathode ray tube. Particles less than 1 nm can be viewed, and since the depth of focus is so much greater than that of the light microscope, information on surface texture can be generated [4].

The understanding and modeling of polymer translocation through a nanopore into a membrane is a difficult task. Nevertheless, in some limitative conditions, Sung and Park [2], Paun [3], Muthukumar [4], and Kong [5], have obtained reasonable results.

The investigation undertaken makes an obvious demarcation between the fractal behavior [6,7] of the polymer and the classical one [8,9,10,11,12], which can be taken into view when estimating an effective translocation time through the membrane [13]. The fractal analysis [14] of the movement of the polymer and its transport through nanometric pores will be the subject of future work.

## 2. Theoretical Background

**Figure 2:**
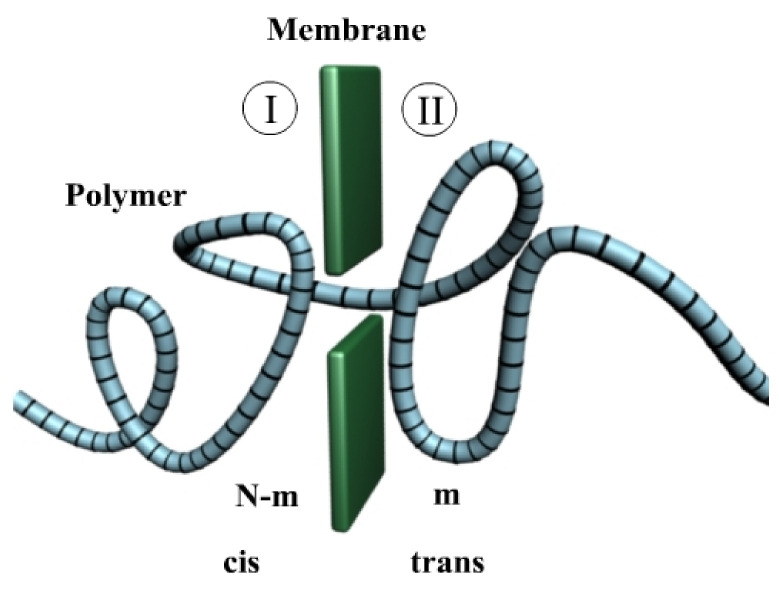
Polymer escape in transition, having *m* monomers in the *trans* area. I is the cis area, while II is the trans area.

We will present the case of an ideal polymer in a 3-dimension extended region separated by a fine boundary named membrane in a free energy barrier attendance [15]. We are supposed to have *m* monomers, which are found in the *trans* area, and *N*-*m* monomers, which are found in the *cis* area, as in Figure 2.

A simplified image of a polymer with *N* monomers in a chain (*N* units), found in the translocation process through a pore of the nanometric waist, a hole disposed in a tridimensional wall.

In the barrier center, it is practiced in a little orifice but wide enough so the monomers from the polymer suite to pass from one side to another.

The related free energy, *F*(*m*), which employs polymer scission/partition function *Z_n_*, will be the following:

(1) $$F\left(m\right)=-k_{B}T\ln Z$$

or, under a chemical potential gradient, [1]

(2) $$F\left(m\right)=-k_{B}T\ln Z+m\Delta \mu$$

Here Δ*μ* = *μ*_1_ − *μ*_2_ is the chemical potential difference on monomer, between the trans and cis region [16].

The scission/partition amount *Z_n_*, in the case of polymeric suite by *n* monomers found in a demi-unbounded domain, into an interaction with a strong barrier and a certain head always fixed at the center of barrier, can be written as follows:

(3) $$Z_{n}\sim n^{\gamma -1}$$

where we can have $\gamma =\frac{1}{2}$ (Gaussian chains), γ=0.69 (self-avoiding chains) and γ=1 (rod-like chains) [13].

To the situation when the partition sum *Z_n_* is equal with $n^{\gamma -1}$ that is in Equation (3), the associated free energy becomes the following:

(4) $$F\left(m\right)=\left(1-\gamma \right)k_{B}T\ln m+m\Delta \mu$$

For the *m* monomers in trans area and *N* − *m* monomers of the polymer in the cis area, the associated free energy is as follows:

(5) $$\beta F_{m}=\left(1-\gamma_{2}\right)\ln\left(m\right)+\left(1-\gamma_{1}\right)\ln\left(N-m\right)m\beta \Delta \mu$$

when the constant terms in sum are not taken into account and $\gamma_{1}$ and $\gamma_{2}$, respectively, are the values of γ in the two regions and $k_{B}T=\beta^{-1}$.

If γ becomes of equal value on either part of the barrier ($\gamma_{1}=\gamma_{2}$),

(6) $$F_{m}=\left(1-\gamma \right)k_{B}T\ln m\left(N-m\right)+m\Delta \mu$$

In conclusion, the free energy of polymer translocation is a function of *m* [17,18]. The second term in Equation (6), mΔμ, enters a linear alteration of border’s baseline.

**Figure 3:**
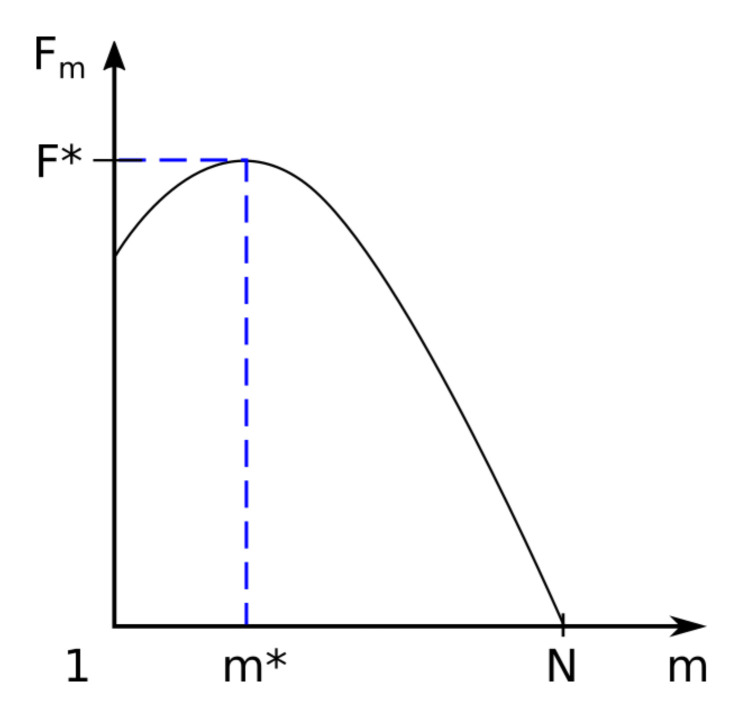
Associated free energy barrier *F_m_* for *m* segments located in *trans* region. The maximum of the function is the value of the function for *m* = m* and is denoted by F* = *F*(m*).

In Figure 3 we have graphically represented the associated free energy barrier for *m* segments located in region II, named trans region (located behind the barrier). Here $F_{m}=F\left(m\right)$, where *m* is the monomers number of the polymer found after the barrier, with 1≤m≤N.

Function *F_m_* admits a maximum at the point where derivative I is annulled, as a function of *m*. The calculations are presented in detail as follows:

(7) $$\frac{dF_{m}}{dm}=\frac{d}{dm}\left(\left(1-\gamma \right)k_{B}T\ln m\left(N-m\right)+m\Delta \mu \right)=\left(1-\gamma \right)k_{B}T\frac{N-2m}{m\left(N-m\right)}+\Delta \mu$$

(8) $$\frac{dF_{m}}{dm}=0\to m^{2}-\left[N+2\frac{\left(1-\gamma \right)k_{B}T}{\Delta \mu }\right]m+\frac{\left(1-\gamma \right)Nk_{B}T}{\Delta \mu }=0$$

The value of *m* for which the first derivative is canceled (equal to zero) is as follows:

(9) $$m=\left[N+2\frac{\left(1-\gamma \right)k_{B}T}{\Delta \mu }\right]\pm \sqrt{{\left[N+2\frac{\left(1-\gamma \right)k_{B}T}{\Delta \mu }\right]}^{2}-4\frac{\left(1-\gamma \right)k_{B}T}{\Delta \mu }}$$

The only positive root is indicated by m* and is equal to the following:

(10) $$m^{*}=\left[N+2\frac{\left(1-\gamma \right)k_{B}T}{\Delta \mu }\right]+\sqrt{N^{2}+4{\left[\frac{\left(1-\gamma \right)k_{B}T}{\Delta \mu }\right]}^{2}}$$

The maximum of the function is the value of the function for *m* = m* and is denoted by $F^{*}=F\left(m^{*}\right)$.

**Figure 4:**
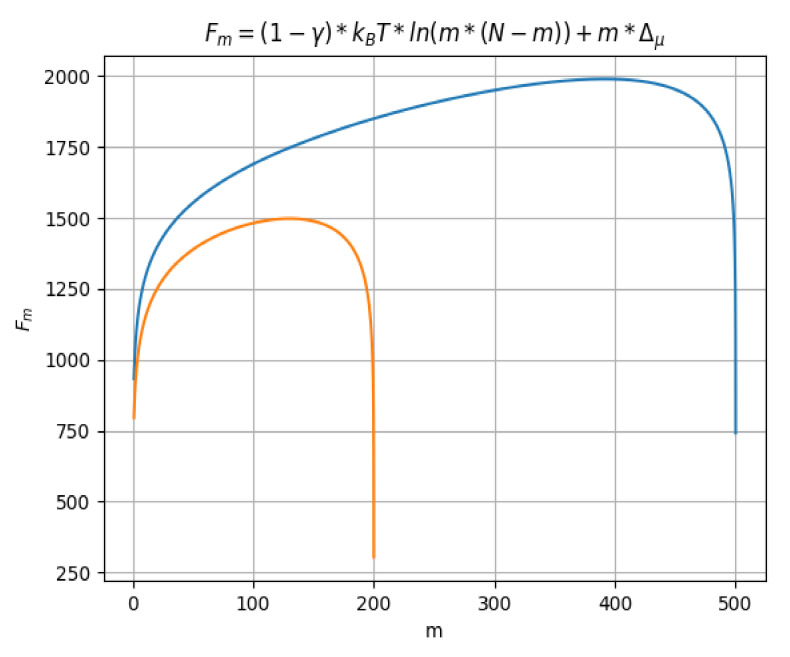
Free energy *F_m_* as function of *m*, for two distinct values taken by *N*.

In Figure 4, *F_m_* (free energy) function of *m*, the segments number located in the region on the right, after the barrier, for the two distinct values taken by *N* (200 and 500) is represented. There are two curves, having two different colors, depending on the maximum value of *N*. Thus, we have the orange color for *N* = 200 and the blue color for *N* = 500.

### Average Time of Polymer Translocation

The average time of polymer translocation to move between cis area and trans area through a nano-pore existent in a fixed solid wall, namely, the escape period, is given by the following equation:

(11) $$\tau =\frac{1}{k_{0}}\int_{n_{1}}^{N}exp\left(F_{m_{1}}\right)dm_{1}\int_{0}^{n_{1}}exp\left(-F_{m_{2}}\right)dm_{2}$$

or depending on the previous formula of the associated free energy *F_m_* = *F*(*m*), we have the explicit integrals for *F*(*m*_1_) and *F*(*m*_2_), as follows:

(12) $$\tau =\frac{1}{k_{0}}\int_{n_{1}}^{N}e^{\left(1-\gamma \right)k_{B}T\ln m_{1}\left(N-m_{1}\right)+m_{1}\Delta \mu }dm_{1}\int_{0}^{n_{1}}e^{-\left(1-\gamma \right)k_{B}T\ln m_{1}\left(N-m_{1}\right)-m_{1}\Delta \mu }dm_{2}$$

Thus, the mean escape period/duration noted *τ* for the polymer lengthiness equal to *Na*, consisting of *N* enchained segments-monomers (each of size *a*), can be found with formula (12), and *k*_0_ is a proportionality constant.

## 3. Materials and Methods

Single-stranded polymer escape time over a membrane nanopore has been well computed using a physicochemical model developed by the authors. To deepen our understanding of the process, we conducted mesoscopic computer simulations to elucidate how polymer chain conformation regulates the dynamic evolution of nanoparticle structures during the transport process of polymer nanocomposites [19].

The polymer translocation process, even of biopolymers, across membranes with nanometric pores is omnipresent in biological systems (such as DNA and RNA escape through nanopores in nuclear chemistry), next to it being protein transport via nano-membrane canal-route and microbe or virus injection into cellular alveolus.

As well supported in a frontier work by Kasianowicz et al. [17], but also based on the results obtained by us in the article recently published in the journal Polymers [13], it was correctly proved that an exterior electric field around the nanopore can lead to single-stranded DNA and RNA particles through the secretion-stuffed (possibly with pure water, that is) of the classic alfa-hemolysin canals.

### The Polymer Escape Probability

As a consequence of the considerations performed by Lubensky et al. [20], we enter the likelihood designated by *P*(*x*, *t*), as a macromolecular chain of lengthiness equal to *x*, to be able to pass by the nanopore in escape period *t*.

The macromolecule has a lengthiness of *L = Na*, where *N* = monomers number of polymer and *a* = monomer length. Initial conditions are related to the fact that at *x* = 0, respective *t* = 0, the polymer head penetrates the nanopore.

The macromolecule probability current density, noted *J*(*x*, *t*), can be represented by the following formula:

(13) $$\frac{\partial P\left(x,t\right)}{\partial t}+\frac{\partial J\left(x,t\right)}{\partial x}=0,$$

a first-order differential equation, with constant coefficients and homogeneous.

In the case of a macromolecule only, it can be accepted that *J* is a linear function of *P* (second-order terms in *P* or more are missing), as follows:

(14) $$J\left(x,t\right)=\nu P\left(x,t\right)-D\frac{\partial P\left(x,t\right)}{\partial x}.$$

The likelihood *P*(*x*, *t*) afterward respects the following equation of diffusion-type having a drift constituent (owed at electromagnetic field presence, for example):

(15) $$\frac{\partial P\left(x,t\right)}{\partial t}=-\nu \frac{\partial P\left(x,t\right)}{\partial x}+D\frac{\partial^{2}P\left(x,t\right)}{\partial x^{2}},$$

where the constant coefficients *D*, and *v* signify the effective diffusion coefficient and mean speed of drift, respectively.

The diffusion Equation (15) can be solved analytically only for cases with simple geometry, constant diffusion coefficients, and simple boundary conditions. Therefore, in analytically untreatable cases, recourse is had to numerical methods (simulations) for solving diffusion equations. Initial and boundary conditions in the most general formulation of diffusion Equation (15) are $P\left(x,t_{0}\right)=P_{0}\left(x\right),\;0<x<L$ and $P\left(0,t\right)=P_{0}^{0}$ respectively $P\left(L,t\right)=P_{L}^{0}$.

The one-dimensional diffusion equation expresses the evolution in time of the probability of the migrant polymer in an infinite flat layer and thickness *L*. Discretization in the *x*-direction of Equation (15) is performed using the Lagrange polynomial of second-degree interpolation, while for time discretization and expression of the derivative, the Taylor series development and the Crank–Nicolson method are used.

Based on the numerical method described above, we proceeded to establish algorithms and program modules. The programming of the algorithms within the modules was performed through a versatile computing code written in C++ programming language. The program was written and tested according to an original procedure developed at this stage, using discrete numerical methods. The testing included both testing the source programs while writing the program and the integrated application.

Note. The discretization for a diffusive process is better behaved with a fully implicit scheme (if the Crank–Nicolson approach is used, one needs to make sure that spurious oscillations in the solution do not develop). We used finite-differences as the iteration method for the solution of boundary-value diffusion problems. The finite-difference method is defined as dimension per dimension, and this makes it easy to increase the “element order” to obtain higher-order accuracy.

Iterative convergence relates to the number of iterations required to obtain residuals that are sufficiently close to zero, either for a steady-state problem or for each time step in an unsteady problem. This error is in addition to the numerical error associated with the truncation error terms.

The magnitude of the difference between the exact value and the approximation is the absolute error, where the relative error is simply the ratio of absolute error to the exact value, which can also be expressed in terms of percentage. To examine the rate of decrease in the relative error, it can be examined the speed of convergence of the algorithm. It is fast with us because we used the known procedure of modified Gauss elimination method.

Mathematically speaking, we are dealing here obviously with the vast-specimen comportment of maximum probability evaluates (MPE’s) of the diffusion process characteristic. Everything is performed by an uninterrupted observation along continuous period (for *t* tends to infinity, t→∞). The triggered effect (according to central limit theorem of a normal distribution for the MPE and the asymptotic chi-squared probability report test) is agreed accurately to standard asymptotic probability results and attends trippingly to the central limit theorem of a stochastic function.

Being in the center of attention the not excessively short polymers, the boundary effects, whereas the polymer is introduced and/or taken out by the nanopore are negligible. The speed *v* is considered as an average speed in time and on the ensemble, as in the Equation (15). Additional interest goes to the casual prevalence of the translocation/escape speed (which translates directly into time of escape/translocation) regarding its mean value *v* and leads to quantifying the diffusion coefficient $D=\underset{t\to \infty }{\lim}\frac{<{\left[x\left(t\right)-x\left(0\right)-vt\right]}^{2}>}{2t}$, where <⋯> represents the average value of the quantity in parentheses, and *x*(0) is the origin position/place of the polymer.

**Figure 5:**
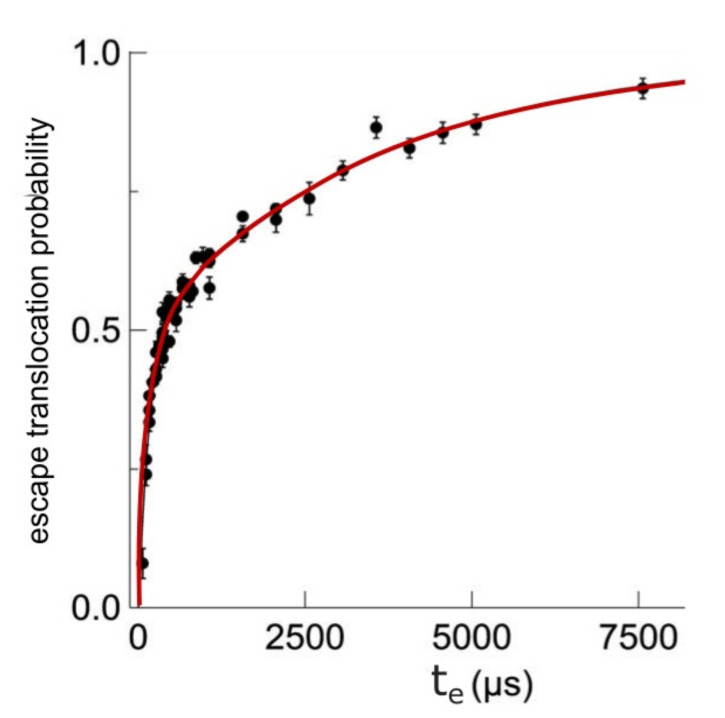
Escape translocation probability as a function of escape time.

**Figure 6:**
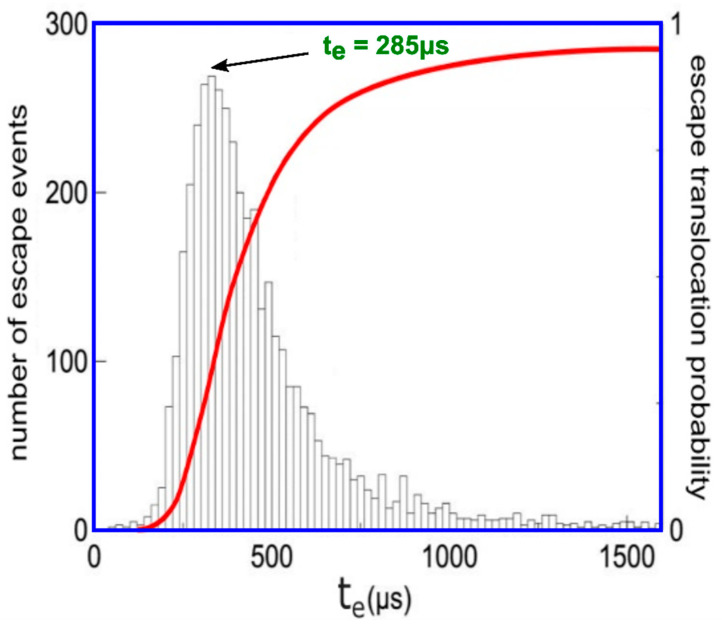
The translocation comportment of three DNA molecules.

Transportation likelihood of DNA through a membrane nanopore called *P_e_*, evidently function of escape time *t_e_*, for time-drive established at 200 μs, is represented in Figure 5 [21]. Every diagrammatic item of data has been estimated from about 1000 cases fulfilled, as pictured in histograms form out of the graphical representation, according to Figure 6.

Error bars (±mean square deviation) situated on every experimental point were calculated by determining the events grouplet/faction in superposed zone between the two real peaks.

The time duration of escape event, *t_e_*, is determined at every passage and the histogram of transport escape length period has been realized out of 5000 passages (principal image of presentation), Figure 6. The longest-lasting likely escape time (top of present histogram repartition) is indicated to be *t_e_* = 285 μs.

## 4. Results Discussions

The model developed above precisely describes the polymer transport process via nanomembranes’ with nanometer-dimensional pores and considers a two-dimensional drift-diffusion phenomenon together with its related familiar formulas [13]. The bio-polymers escape-translocation procedure by the membranes’ small waist orifices is refound throughout biological complex structures, if we think primarily of nucleic acids (DNA and RNA) transport across holes at a nanometric level, or even of protein driven across membrane canals (the so-called inland waterways), along with the microbial germs inoculation in living cellular artifacts.

**Figure 7:**
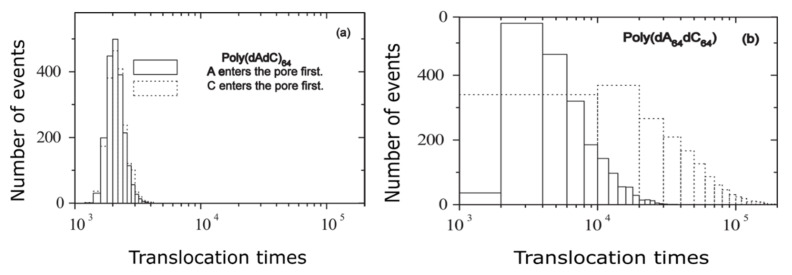
Translocation times histogram: (**a**) poly(dAdC)_64_ and (**b**) poly(dA_64_dC_64_) under *F* = 0.5 [13].

It has been measured the escape time of polymer translocation process via nanomembrane for two distinguished nucleic acids of the same extension/dimension, but with a configuration variable existing just in the itemized order construction. In Figure 7, two reference examples are presented, such as the histograms of testing of hetero-DNAs for (a) poly (dAdC)_64_ and (b) poly (dA_64_dC_64_), respectively.

Because the chemical potential difference Δμ controls the escape time τ, the limiting situation for Δμ can lead to the same simple analytical formulas for τ [22].

For example, in the Δμ=0 case and symmetric barriers, the solution of the Equation (6) is $\tau =\alpha N^{2}$, where α depends on $k_{0},$ $\gamma_{1}$ and $\gamma_{2}$. In the particular case $\gamma_{1}=\gamma_{2}=\frac{1}{2}$ we have $\tau =\frac{M^{2}}{16k_{0}}N^{2}$. By combining the two examples above, we obtain the following result:

(16) $$\tau =\left\{\begin{array}{c} \alpha N^{2},\left|\Delta \mu \right|=0\\ \frac{N^{2}}{16k_{0}},\left|\Delta \mu \right|=0\;and\;\gamma_{1}=\gamma_{2}=\frac{1}{2} \end{array}\right.$$

In other words, in the absence of the potential (Δμ=0), the translocation time has a square behavior, $\tau \sim N^{2}$.

Sung and Park regard the polymer translocation as a stochastic phenomenon, in which a solid obstacle placed in front of the polymer is crossed. Polymer escape duration now τ becomes the average first passage period (average escape time) of the diffusion process between two extreme numbers of segments n=0 and n=N, [2].

The results, in the case when *μ*_1_ = *μ*_2_, are the following:

(17) $$\tau =\frac{M^{2}}{16}\frac{L^{2}}{D}\sim L^{3}\;(\mathrm{flexible}\;\mathrm{chain})$$

and, in the case of a rigid rod, where $F\left(m\right)=0$,

(18) $$\tau =\frac{L^{2}}{2D}\sim L^{3}\;(\mathrm{rigid}\;\mathrm{rod}).$$

In the above equations, *D* signifies diffusion coefficient subject to scaling, more precisely *D*~*L*, where *L* represents chain profile lengthiness (a polymer length).

As Muthukumar [4] affirms, the difference in the exponent for the *N*-dependence of τ between the two models is the dynamics of translocation and the nature of $k_{0}$, while the formalism is exactly the same.

When the entropic terms are weak compared to the third term on the right-hand side mβΔy in Equation (4) and for a favorable chemical potential gradient (Δy<0), τ is given by the following:

(19) $$\tau =\frac{N}{k_{0}}\frac{k_{B}T}{\left|\Delta y\right|}\left\{1-\frac{k_{B}T}{N\left|\Delta y\right|}\left[1-e\alpha_{P}\left(-\frac{N\left|\Delta y\right|}{k_{B}T}\right)\right]\right\}\sim L^{3}$$

with the limits

(20) $$\tau =\left\{\begin{array}{c} \frac{k_{B}T}{k_{0}\left|\Delta y\right|}N,N\left|\Delta y\right|>1\\ \frac{N^{2}}{2k_{0}},N\left|\Delta y\right|<1 \end{array}\right.$$

In addition, the conditions equivalent from a physical point of view to the situation in which the drift term dominates the transport problem are as follows:

(21) $$\tau \sim N^{2},\;\mathrm{for}\;N\left|\Delta \mu \right|<1.$$

In the case of polymer translocation against the chemical potential gradient, the escape time *τ* can be expressed by exp (*N*) for a large polymer size.

**Figure 8:**
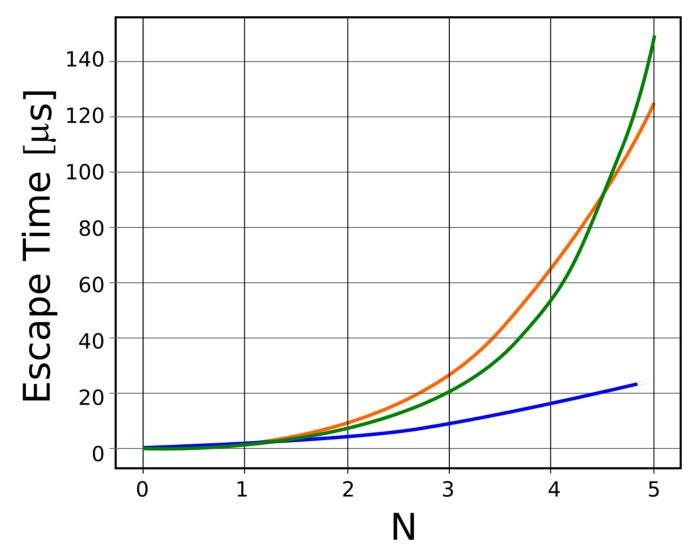
Escape translocation time (μs), for three distinct equations that function of *N*.

In Figure 8, escape translocation time functions of *N* for three distinct mathematical equations, drawn in three different colors, are shown. Thus, the blue color represents the variation as a function of *N*^2^, the red color represents the variation as a function of *N*^3^, and the green color represents variation as a function of exp (*N*).

*Note.* Today, transport accelerators of a polymer through nanometric pores are used, among which we notify the nanoparticle-assisted polymer translocation through a nanopore. Thus, the translocation time decreases consistently, which makes any of the calculation formulas be multiplied by a subunitary coefficient [23].

*Remarks.* Theoretical and simulation results obtained from calculations made by us are in splendid accord with the data obtained experimentally by valuable authors in the works placed in the bibliography [24,25].

A linear dependence of the most probable time, *t_p_*, with *N* has been experimentally verified for DNA molecules larger than 12 nucleotides [26,27].

Thus, even more, Slonkina and Kolomeisky [28] have made an improvement to it by using a tierce area, which can take into account the finite depth of an ideal membrane and the real orifice dimension, but also polymer translocation under a pulling force. It is worth considering [29,30].

In the last period, more precisely in the year 2022, flow-induced translocation of linear and ring polymers has been studied using a combination between multiparticle collision dynamics and molecular dynamics concepts [31], but also Langevin dynamics (LD) simulations [32].

## 5. Conclusions

In the current paper, a model for polymer transport through membranes with nanometric pores is developed.

The news produced in the article is related to the following aspects: The way of solving transport equations, which is a direct and modern one, and especially the boundary conditions, are the novelties brought here. They may be more relevant by obtaining improved solutions that are much simpler and easier to represent graphically.

As a success of this paper, we mention that we applied these equations to a new spectrum of polymers, especially organic, natural, even DNA, etc.

An important result is the estimation of the macromolecule escape duration, which is the time to accomplish the polymer translocation between the left part and the right region of the barrier, where it is found entirely at the end of the process.

In the case of unsymmetrical membranes but of large polymeric chain size, in the strong-negative-bias limit $\left(\Delta \mu \ll 0\right)\tau$ we will have a linear dependency of monomer number total in the polymer, ~*N*.

Although the model presented in this study is an idealistic theoretical model, it leads to correct particular results without discussion.

The theoretical estimations are functions of the polymer size and make a distinction thus between polymer waist, long polymer, and short polymer, respectively. The detailed model presented here is confirmed by experimental results.

The transport time duration for all escape events, named *t_e_*, has been determined at every polymer transition, and the long time period histogram of individual processes has been realized out of 5000 passages. The longest-lasting likely escape time (noted at the top of the present histogram repartition) is determined to be *t_p_* = 330 μs.

The thickness dimension of the pore dictates the classification of polymers into the following two obvious categories: “long” polymers, the first demarcation, and “short” polymers, the second demarcation.

## Data Availability

The data used to support the findings of this study cannot be accessed due to commercial confidentiality.
